# Natural Killer Cell Assessment in Peripheral Circulation and Bronchoalveolar Lavage Fluid of Patients with Severe Sepsis: A Case Control Study

**DOI:** 10.3390/ijms18030616

**Published:** 2017-03-12

**Authors:** Paulo Souza-Fonseca-Guimaraes, Fernando Guimaraes, Caroline Natânia De Souza-Araujo, Lidiane Maria Boldrini Leite, Alexandra Cristina Senegaglia, Anita Nishiyama, Fernando Souza-Fonseca-Guimaraes

**Affiliations:** 1 Hospital Universitário Cajuru—Pontifícia Universidade Católica do Paraná—Curitiba PR, Curitiba, Paraná CEP 80050-350, Brazil; paulosfguimaraes@gmail.com; 2 Departamento de Fisiologia, Setor de Ciências Biológicas, Universidade Federal do Paraná (UFPR), Curitiba, Paraná CEP 81531-990, Brazil; 3 Hospital da Mulher “Prof. Dr. José Aristodemo Pinotti”—CAISM, University of Campinas, São Paulo CEP 13083-887, Brazil; fernando@caism.unicamp.br (F.G.); caroline.natania@yahoo.com.br (C.N.D.S.-A.); 4 Núcleo de Tecnologia Celular—Pontifícia Universidade Católica do Paraná—Curitiba PR, Curitiba, Paraná CEP 80215-901, Brazil; lidiane.leite@pucpr.br (L.M.B.L.); alexandra.senegaglia@pucpr.br (A.C.S.); 5 Faculty of Medicine, Dentistry and Health Sciences, University of Melbourne, Melbourne, Victoria 3010, Australia; 6 Division of Molecular Immunology, The Walter and Eliza Hall Institute of Medical Research and Department of Medical Biology, University of Melbourne, Parkville 3052, Australia

**Keywords:** natural killer (NK) cells, lung sepsis, lymphopenia, bronchoalveolar lavage (BAL)

## Abstract

Sepsis is a complex systemic inflammatory syndrome, the most common cause of which is attributed to systemic underlying bacterial infection. The complete mechanisms of the dynamic pro- and anti-inflammatory processes underlying the pathophysiology of sepsis remain poorly understood. Natural killer (NK) cells play a crucial role in the pathophysiology of sepsis, leading to exaggerated inflammation due their rapid response and production of pro-inflammatory cytokines such as interferon gamma (IFN-γ). Several studies have already shown that NK cells undergo lymphopenia in the peripheral blood of patients with sepsis. However, our understanding of the mechanisms behind its cellular trafficking and its role in disease development is restricted to studies in animal models. In this study, we aimed to compare the human NK cell subset (CD56^bright or dim^) levels in the peripheral blood and bronchoalveolar lavage (BAL) fluid of sepsis patients. We conducted a case-control study with a sample size consisting of 10 control patients and 23 sepsis patients enrolled at the Hospital Cajuru (Curitiba/PR, Brazil) from 2013 to 2015. Although we were able to confirm previous observations of peripheral blood lymphopenia, no significant differences were detected in NK cell levels in the BAL fluid of these patients. Overall, these findings strengthened the evidence that peripheral blood lymphopenia is likely to be associated with cell death as a consequence of sepsis.

## 1. Introduction

Sepsis is defined as life-threatening organ dysfunction caused by dysregulated host responses to infection (as per the Third International Consensus definition for sepsis and septic shock) [1,2]. Currently, sepsis is the tenth leading cause of death in high-income countries, accounting for more than 210,000 annual deaths in the USA [3]. With an increasing incidence of sepsis of approximately 9% each year and an increasing emergence of antibiotic resistance in microorganisms, which worsens the efficacy of antibiotic treatment against sepsis [3,4], it is of great urgency to develop a more targeted and efficient treatment strategy against sepsis. Sepsis is defined as the development of two or more symptoms of systemic inflammatory response syndrome (SIRS) including abnormal thermal regulation, atypical leukocyte count, tachycardia and rapid breathing [5,6]. Left untreated, sepsis may progress to severe sepsis with the development of further complications such as multiple organ failure (MOF) [6]. These patients have an increased risk of subsequently advancing into septic shock—a state of persistent hypotension despite intravenous fluid resuscitation due to acute circulatory failure. Both MOF and septic shock lead to eventual death [5,6]. Despite being extensively studied, the pathogenesis of sepsis and the contribution of cellular mediators of the inflammatory response remain poorly understood.

Recent studies have revealed that the pathogenesis of sepsis is not merely a result of an exacerbated host inflammatory response in an attempt to clear invading pathogens. Combined dysregulation of both pro- and anti-inflammatory responses of the host results in an overzealous release of cytokines and chemokines known as the “cytokine storm”, resulting in the initial stage of SIRS [7,8,9]. Although a localized inflammatory response is needed for the clearance of infection, failure of anti-inflammatory mediators to control the inflammatory process can lead to endothelial dysfunction allowing further invasion of pathogens into host organs [6]. In addition, patients surviving the initial SIRS stage were discovered to frequently enter an immunosuppressive state during the later stage of sepsis, described as a compensatory anti-inflammatory response syndrome (CARS) [10,11]. CARS is a consequence of the body’s initial pro-inflammatory immune state during sepsis, leading to a defective adaptive immune response rendering patients susceptible to secondary nosocomial infections responsible for their death [12].

The innate immune system is a key player in the early stages of sepsis and various studies have focused on the role of monocytes and macrophages in the pathogenesis of sepsis [13]. Natural killer (NK) cells were initially described as innate lymphocytes with the ability to induce targeted cell death in tumor cells and virus-infected cells by secreting granzymes (Grz) and perforin (PFP) [14,15,16]. The action of NK cells is regulated via activating and inhibitory receptors, which further divide NK cells into different subtypes with different effector functions which have not yet been completely described [17]. Apart from mediating cytotoxicity, NK cells have also been shown to have regulatory functions on inflammation due to their ability to engage in reciprocal crosstalk with other immune cells such as dendritic cells which provide the cytokine microenvironment required for NK cell functions. This crosstalk is crucial in determining the response of NK cells, which subsequently act on the development of the downstream immune response against infection by suppressing or amplifying inflammation. NK cells produce an array of cytokines such as IFN-γ, GM-CSF and TNF-α and chemokines such as CCL1, CCL2 and CCL5, which contribute to pathogen clearance but also amplify the inflammatory response [17,18]. In addition, recent discoveries have implicated the contribution of NK cells in the pathogenesis of sepsis due to their expression of innate sensors against pathogen-associated molecular patterns (PAMPs) and damage-associated molecular patterns (DAMPs), enabling them to directly recognize and respond to various pathogens [14,17].

Clinical research has identified that the number of circulating NK cells in the bloodstream correlates inversely with patient survival, with significantly lower circulating NK cell numbers in survivors of sepsis [19]. Additionally, the depletion or priming deficiency of NK cells in experimental murine models of sepsis conferred greater survival benefits against sepsis [20,21,22]. This evidence strongly suggests that NK cells participate in sepsis, and their circulating levels may be a biomarker of mortality. The molecular mechanism by which NK cells participate in the pathogenesis of sepsis has not been fully elucidated, and whether peripheral blood lymphopenia is linked to a rise in NK cell levels in the infection sites has not being studied in patients. Here, we aimed to further study NK cell–related lymphopenia with their respective levels in the bronchoalveolar lavage (BAL) fluid of lungs of infected patients maintained in the intensive care unit (ICU), and observed for the first time that the lymphopenia in peripheral blood is not associated with increased NK cell levels in the BAL fluid.

## 2. Results

### 2.1. Patients Characteristics

Twenty-three patients who were characterized as having severe sepsis had evidence of primary infection in the lungs, and displayed clinical criteria of sepsis (fever or hypothermia, tachycardia (heart rate > 90 bpm), tachypnea (respiratory rate > 20 mpm), leukocytosis (>12,000) or leukopenia (<4000), BA stenosis (>10%)), or an increased sequential organ failure assessment (SOFA) score (two points or more) associated with radiographic imaging identified by tomography and/or thorax radiography, suggesting pulmonary damage due to infection. Patient characteristics are described in Table 1. Healthy controls were patients who have previously undergone or have a currently established tracheostomy, consisting of 10 trauma survivor patients (e.g., following a cerebrovascular accident, cranioencephalic trauma, or polytrauma). These patients had prolonged orotracheal intubation until complete recovery, and displayed no symptoms of infection/pneumonia or lung damage during the biopsy sampling. Although healthy controls were subjected to peripheral blood and BAL fluid sampling, most of the parameters displayed by these patients were not applicable for these controls. The sepsis patients have all undergone mechanical ventilation and most had evidence of primary infection in the lungs (82.61% with confirmed infection from the tracheal aspirate). Fifteen patients had Gram-negative bacterial infections, while two displayed Gram-positive bacterial infections, and three had fungal infections and three had undetermined microbial infections.

### 2.2. Pro-Inflammatory Cytokine Profiling in Bronchoalveolar Lavage (BAL) Fluid Samples after Sepsis

**Figure 1:**
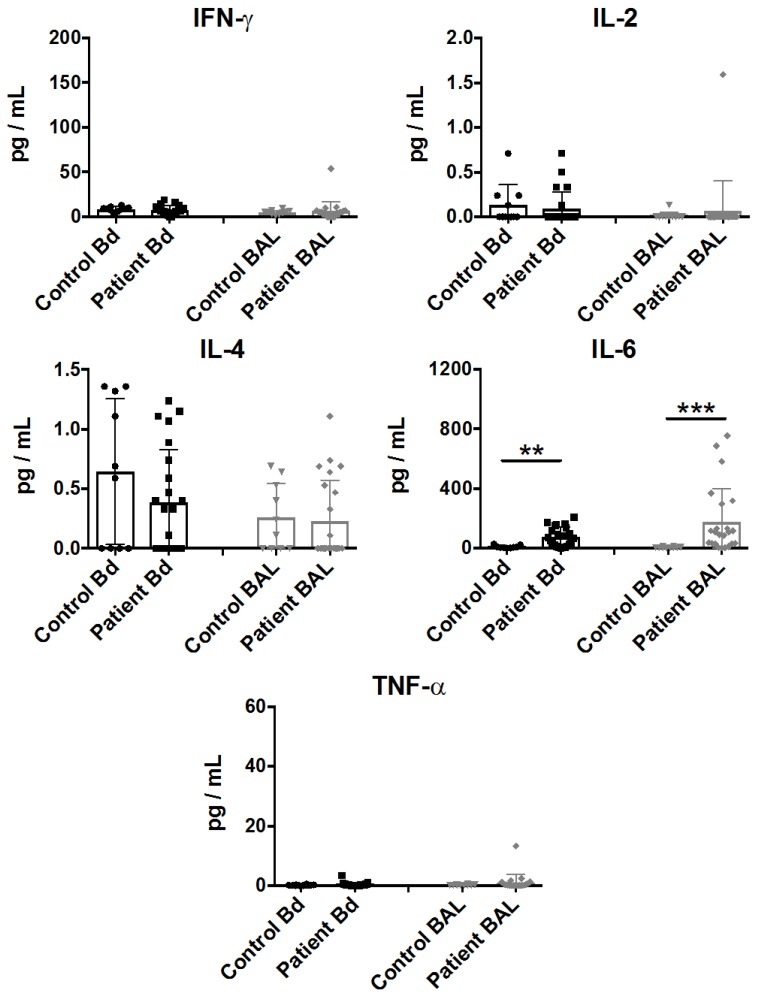
Cytokine profiling of plasma (represented by Bd) and bronchoalveolar lavage (BAL) fluid from sepsis patient samples. Cytokine assessment was performed on plasma or BAL fluid samples which were collected within 48 h of clinical classification of severe sepsis and ICU admission. IFN-γ, IL-2, IL-4, IL-6, and TNF-α levels are represented as pg/mL in mean ± SEM, with all data from individual controls or patients. Statistical analysis was performed using the Mann-Whitney test, where ** *p* < 0.01, or *** *p* < 0.001 were considered for statistical significance.

Multiple cytokine patterns were associated with a systemic response in sepsis [23]. To characterize the pro-inflammatory cytokine content in the BAL fluid and in peripheral blood, we screened the samples with a customized Th1/Th2 cytokine detection array. Consistent with previous studies [24], IL-6 was significantly upregulated in the plasma samples from sepsis patients, as well as in the BAL fluid samples, while minimal and insignificant detection of IL-2 and IL-4 was observed (Figure 1). In contrast, we failed to observe a significant increase in other pro-inflammatory cytokines such as IFN-γ and TNF-α in these clinical settings.

### 2.3. Natural Killer (NK) Cell Subsets Are Reduced in Peripheral Blood but Unchanged in BAL Fluid Samples after Sepsis

**Figure 2:**
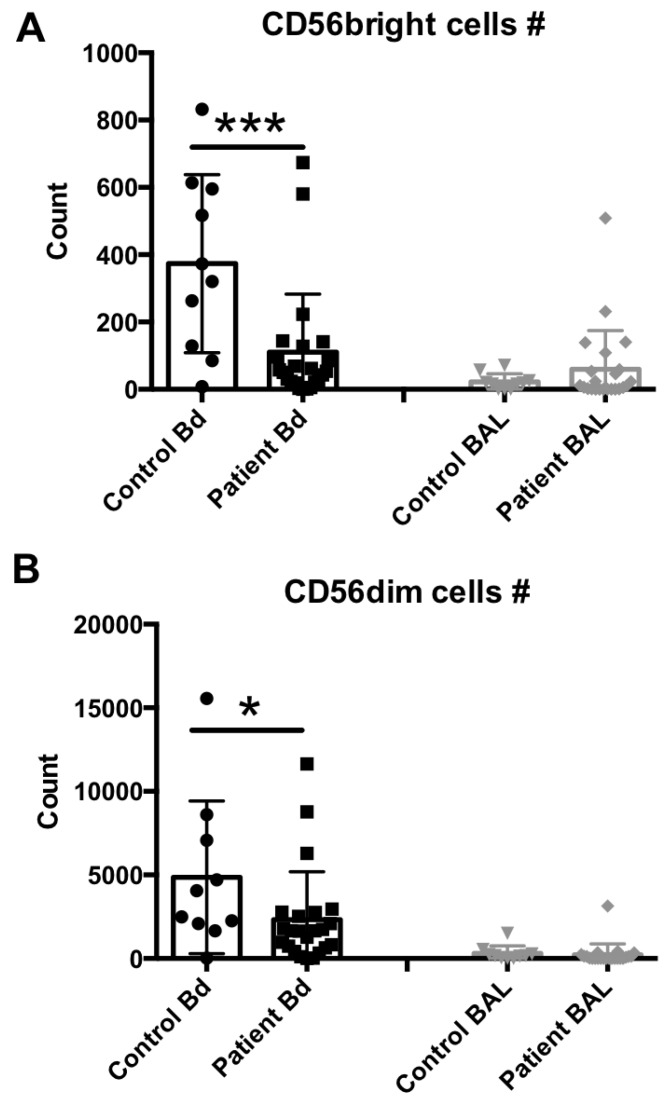
NK cell subset assessment in the peripheral blood or BAL fluid of sepsis patients. (**A**) Peripheral blood (number per 50 μL blood) and BAL fluid (number recovered from the total BAL lavage) CD56^bright^, CD3^neg^, 7AAD^neg^ cells were quantified by flow cytometry. (**B**) Peripheral blood (number per 50 μL blood) and BAL fluid (number recovered from the total BAL lavage) CD56^dim^, CD3^neg^, 7AAD^neg^ cells were quantified by flow cytometry. Statistical analysis was performed by one-way ANOVA followed by the Tukey post hoc test, where * *p* < 0.05, or *** *p* < 0.001 were considered for statistical significance.

We next assessed the lymphocytic composition of these samples. The numbers of CD3^neg^ and CD56^bright^ or CD56^dim^ NK cell subsets in the peripheral blood and BAL fluid samples from sepsis patients and healthy controls were determined and compared. Representative flow cytometry results from each group are shown in Supplementary Figure S1, and the median and scattering plot comparisons of each group are shown in Figure 2A (for CD56^bright^ subset) and 2B (for CD56^dim^ subset). In agreement with previous studies [19,25,26], both NK cell subsets were clearly reduced in number in the peripheral blood compartment of sepsis patients, in comparison to healthy controls. In contrast, no differences were observed in these cell levels in the BAL fluid samples.

### 2.4. T CD4 and T CD8 Cell Levels Are Also Reduced in the Peripheral Blood, but Not in the BAL Fluid of Sepsis Patients

**Figure 3:**
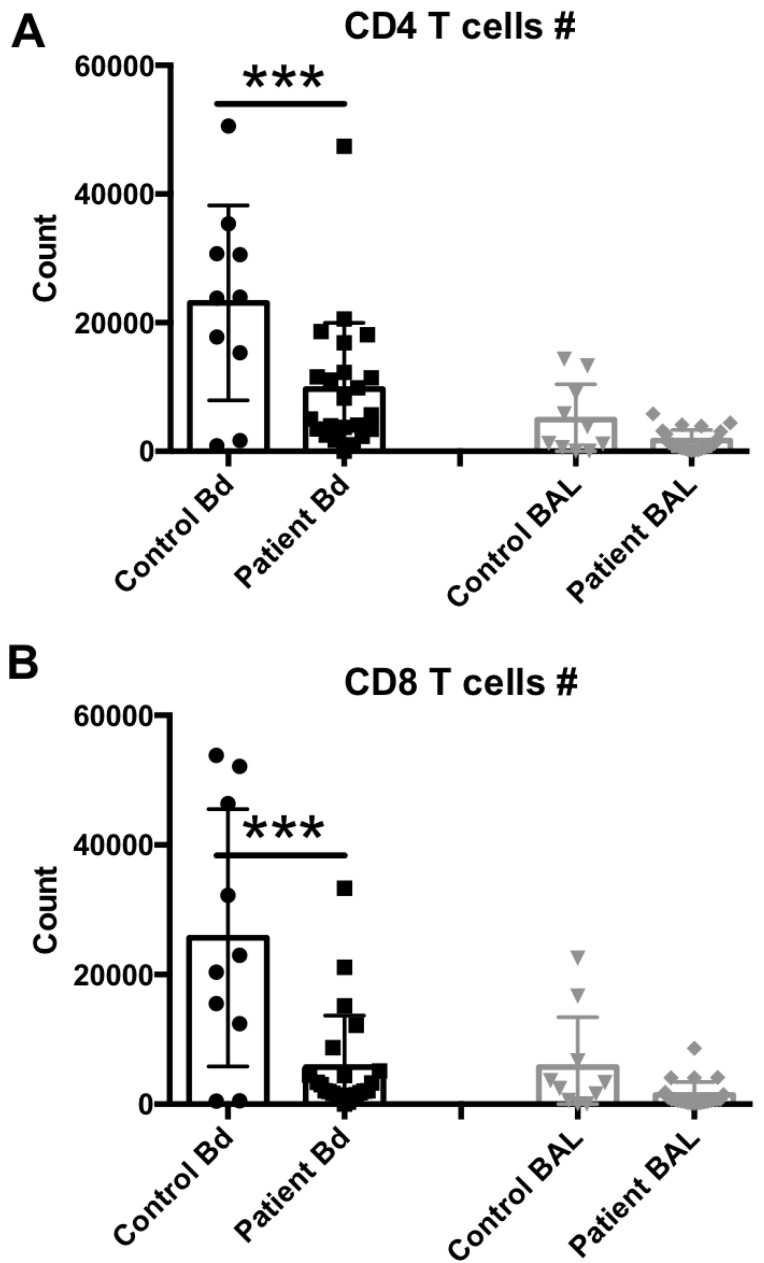
T cell subset assessment in the peripheral blood or BAL fluid of sepsis patients. (**A**) Peripheral blood (number per 50 μL blood) and BAL fluid (number recovered from the total BAL lavage) CD4^+^, CD3^+^, CD8^neg^, 7AAD^neg^ cells were quantified by flow cytometry. (**B**) Peripheral blood (number per 50 μL blood) and BAL fluid (number recovered from the total BAL lavage) CD4^neg^, CD3^+^, CD8^+^, 7AAD^neg^ cells were quantified by flow cytometry. Statistical analysis was performed by one-way ANOVA followed by the Tukey post hoc test, where *** *p* < 0.001 was considered for statistical significance.

To compare whether reduced NK cell levels in the peripheral blood and sustained NK cell levels in the BAL fluid are comparable with other lymphocytic subset levels, also described as lymphopenic in sepsis [25], we performed additional staining to determine T CD4 and T CD8 cell levels in the same samples. Representative flow cytometry results from each group are shown in Supplementary Figure S2, and the median and scattering plot comparisons of each group are shown in Figure 3A (for T CD4 cell subset) and Figure 3B (for T CD8 cell subset). In agreement with the NK cell levels, other lymphocytes such as T cells also seem to suffer from the global lymphocytic lymphopenia previously observed in the peripheral blood of sepsis patients. However, the BAL fluid levels also remained unchanged for these cell subsets, suggesting that the lung microenvironment does not mimic the lymphopenia observed in the peripheral circulation or recruit lymphocytes in response to infection.

## 3. Discussion

Our current study aimed to further characterize the immune status of NK cells in both the peripheral blood and the BAL fluid of patients with bacterial pneumonia. Consistent with previous reports from our group and others [25,26], we observed both NK and T cell lymphopenia in the peripheral blood of sepsis patients. The site of infection as well as both systemic and local variables were historically considered in scores that predict patient survival [27]. Sample acquisition of peripheral blood for studying immune cells in sepsis patients is commonly performed due to the convenience of acquiring these types of samples. However, due to the nature of the bacterial insult, the type of leukocytes found at the site of infection may be significantly different from the peripheral blood cells due to the complex and compartmentalized inflammatory response in sepsis [28]. In addition, a recent study demonstrated that the local microenvironment controls the compartmentalization of NK cell responses during experimental systemic inflammation [9]. To date, our study was the first to compare these cell populations simultaneously in both peripheral blood and BAL fluid samples from sepsis patients, and to measure these cell levels systemically and at respective infection sites. Interestingly, both NK cells and other lymphocytes such as T CD4 and T CD8 cells displayed blood lymphopenia, while no altered levels of these cell subsets were observed in the BAL fluid samples.

Lymphopenia during sepsis is a clinically described phenomenon which is hypothesized to result either because of trafficking to the infection sites, or cell death. Hotchkiss and colleagues previously demonstrated that lymphopenia is a consequence of lymphocyte apoptosis during sepsis, and that prevention of lymphocyte death improves survival in mice [29]. The same group also reported that administration of interleukin-(IL-)7 and IL-15 reversed lymphocyte apoptosis and improved sepsis survival in experimental mice models [30,31]. The same group also showed that in patients, persistent lymphopenia is a predictor of mortality and sepsis-induced immunosuppression [32]. Based on these findings, new clinical trials studying the administration of recombinant IL-7 are currently being evaluated in an attempt to reverse lymphopenia and sepsis-induced immunosuppression in sepsis patients (ClinicalTrials.gov identifier: NCT02640807 and NCT02797431). In contrast to the cell death–induced lymphopenia hypothesis, Herzig and colleagues demonstrated that in experimental peritoneal sepsis, murine NK cells migrate into the peritoneal cavity via a chemokine (C-X-C motif) receptor 3 (CXCR3)-induced mechanism, resulting in peripheral blood lymphopenia. This suggests that it may be a consequence of chemotaxis to the infection site [33], as well as the inflammation/cytokine-induced activation state of both lymphocytes and endothelial cells [34]. One possible hypothesis, which may explain peripheral blood lymphopenia but unchanged cell levels in other compartments, is that cellular activation may result in increased cell adhesion molecules which anchor specific lymphocytes to the endothelial wall. This possibility could result in less soluble lymphocytes in blood, which would resemble a peripheral blood lymphopenia scenario.

Although experimental sepsis is a powerful tool used to understand the mechanisms of immune responses in the process of this syndrome, the immune response variability to pathogen-associated molecular patterns (PAMPS) between different species can vary by over 10,000-fold between mice and humans [35]. In addition, a subsequent systematic study which evaluated how well murine models mimic human sepsis revealed poor translation of genomic responses between species [36]. Our study supports the hypothesis that lymphopenia in sepsis is likely to be associated with apoptosis-induced cell death during the exaggerated inflammatory event [37]. However, our study was limited to BAL fluid sampling, and there is a possibility that using samples from homogenates of lung biopsies could reveal a different outcome. In this study, we further extended the concept of NK cell levels in different compartments in sepsis patients, and demonstrated that blood lymphopenia is not necessarily associated with increased levels of these cells in the BAL fluid of lung-related sepsis patients.

## 4. Experimental Section

### 4.1. Clinical Samples

Intensive care unit (ICU) patients with a confirmed diagnosis of pneumonic sepsis, and healthy controls undergoing post surgery-related tracheostomy monitoring, were recruited into an established, ethically-approved study investigating the natural history and pathogenesis of sepsis in the Hospital Cajuru, Curitiba/PR, Brazil. The study was approved by the regional ethics committee of the Hospital Cajuru and Pontifícia Universidade Católica do Paraná, Curitiba/PR, Brazil (CAAE: 16930013.0.0000.0102, Number: 439.881, Date of approval: 9 October 2013), and Federal University of Parana, Curitiba/PR, Brazil (Number: 398.997, Date of approval: 11 September 2013). In addition, a written informed consent to participate in the study was obtained for each patient, or if not possible, from the patient’s next-of kin. Peripheral blood and BAL fluid samples were acquired from 23 patients within 48 h post ICU admission. Patients were recruited according to sepsis classification, where any subject with cancer, HIV, undergoing tissue transplantation or chronic corticosteroid or other immunosuppressive medication therapy were excluded. Healthy controls undergoing prolonged orotracheal intubation were monitored by routine bronchoscopy to survey lung status. BAL fluid was collected, as previously described [38,39], from segmental bronchus from medium lobe, excluding any subject with active lung disease at the time of the exam. BAL fluid samples were promptly placed in silicone tubes connected to the aspiration system through the vacuum suction system. Blood samples were also acquired from the same patients and healthy controls’ peripheral veins in heparin-containing collection tubes. All samples were immediately sent for analysis.

Individuals with confirmed bacterial infection were classified as patients with sepsis. Individuals in whom infection was not confirmed were classified as Systemic Inflammatory Response Syndrome (SIRS) patients and were not included in the analysis. Patient samples were compared to samples from 10 healthy volunteers and all samples were collected at the Hospital Cajury (Serviço de Endoscopia Respiratória e Cirurgia Torácica do Hospital Universitário Cajuru), Curitiba/PR, Brazil. All fresh samples were processed for flow cytometry, and cytokine analysis was performed from frozen BAL supernatant and plasma, maintained at −80 °C until the day of analysis.

### 4.2. Reagents

Labeled antibodies against the following antigens were used: anti-CD3 (clone UCHT1), anti-CD56 (clone HCD56), anti-CD19 (clone HIB19), anti-CD20 (clone 2H7), anti-CD4 (clone RPA-T4), anti-CD8 (clone SK1). All antibodies, Fc block reagent (anti-CD16/32) and 7-AAD, used as viability dye, were purchased from Biolegend (San Diego, CA, USA). Cytokines were measured by flow cytometry using the Human Th1/Th2 Cytometric Bead Array Kit (BD Bioscience, San Jose, CA, USA).

### 4.3. Flow Cytometry

Cell surface antigens were labeled by diluting antibodies in staining buffer at the concentration suggested by the manufacturers. All samples were processed as previously described by our group [26]. All flow cytometry data was acquired via a FACS Calibur (BD Biosciences, San Jose, CA, USA), and analyzed using the Flow Jo software (Tree Star, Ashland, OR, USA). Cell numbers were determined as previously described [26], by gating the respective lymphocyte subsets using the FACS Calibur cytometer, allowing for absolute counting. All cytokines from sepsis patients and healthy control samples were detected using Cytometric Bead Array (CBA) technology according to the manufacturer’s instructions using a FACS Verse cytometer to acquire samples, and FCAP Array software to analyze them (BD Biosciences, San Jose, CA, USA).

### 4.4. Statistical Analysis

Statistical analysis was achieved using Graph Pad Prism Software (GraphPad, La Jolla, CA, USA). Data was considered to be statistically significant where the *p*-value was equal to or less than 0.05. Statistical tests used were the Mann Whitney test, or one-way ANOVA with Tukey’s post hoc test for group comparison.

## Figures and Tables

**Table 1:** Parameters of the studied patients.

Parameter	Controls (*n* = 10)	Sepsis (*n* = 23)
Age mean (SD)	49.4 (22.6)	59.17 (17.02)
Gender, *n* (%)		
Male	8 (80%)	16 (69.57%)
Female	2 (20%)	7 (30.43%)
Body mass index, kg/m^2^, mean (SD)	28.13 (2.48)	26.14 (4.61)
Comorbidity, *n* (%)		14 (60.86%)
Hemorrhagic cerebrovascular accident	N/A	4 (17.39%)
Other cerebrovascular accident (ischemia, or aneurysm)	4 (40%)	3 (13.04%)
COPD	N/A	1 (4.35%)
Cancer	N/A	1 (4.35%)
Lung	N/A	4 (17.39%)
Liver failure	N/A	1 (4.35%)
Skull/spine fracture	N/A	4 (17.39%)
Polytrauma	6 (60%)	4 (17.39%)
Mechanic ventilation	N/A	23 (100%)
Infection site		
Urinary tract	N/A	10 (43.48%)
Tracheal aspirate	N/A	19 (82.61%)
Mechanic ventilation	N/A	23 (100%)
Leukocytes, %/mm^3^, mean (SD)	N/A	15,059.56 (10424.43)
SOFA, mean (SD)	N/A	5.43 (2.27)
SAPS II, mean (SD)	N/A	21 (9.95)
Length of stay in hospital, days, means (SD)	N/A	39.82 (21.21)
Length of stay in ICU, days, mean (SD)	N/A	18.78 (9.96)
In-hospital mortality number (%)	N/A	7 (30.43%)
Gram-positive bacteria (%)	N/A	2 (8.69%)
Gram-negative bacteria (%)	N/A	15 (65.21%)
Fungus	N/A	3 (13.04%)
Undetermined microbial infection	N/A	3 (13.04%)

COPD, chronic obstructive airways disease; N/A, not applicable, SOFA, sequential organ failure assessment; SAPS, simplified acute physiology score.

## Supplementary Materials

Supplementary materials can be found at www.mdpi.com/1422-0067/18/3/616/s1.
