# Supplementary material for: Natural Killer Cell Assessment in Peripheral Circulation and Bronchoalveolar Lavage Fluid of Patients with Severe Sepsis: A Case Control Study
Source: Int J Mol Sci. 2017 Mar 12;18(3):616. doi: 10.3390/ijms18030616 (PMC5372632; doi:10.3390/ijms18030616)

Supplementary Materials: Natural Killer Cell Assessment in Peripheral Circulation and Bronchoalveolar Lavage Fluid of Patients with Severe Sepsis: A Case Control Study

Paulo Souza-Fonseca-Guimaraes, Fernando Guimaraes, Caroline Natânia De Souza-Araujo, Lidiane Maria Boldrini Leite, Alexandra Cristina Senegaglia, Anita Nishiyama and Fernando Souza-Fonseca-Guimaraes

Supplementary Figure 1

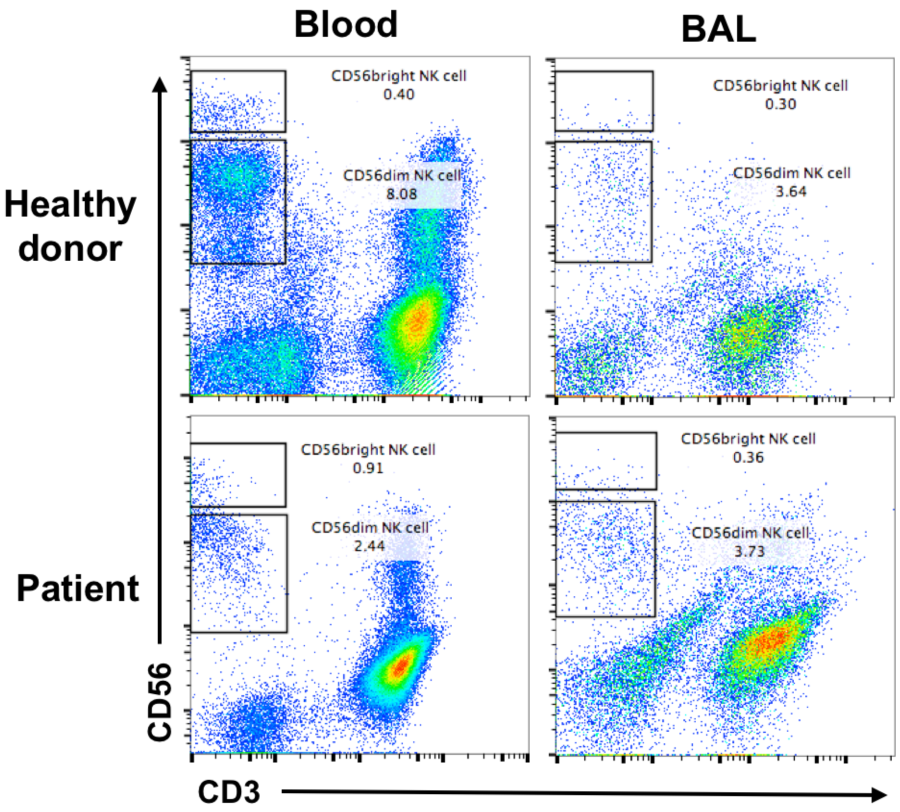

Supplementary Figure 2

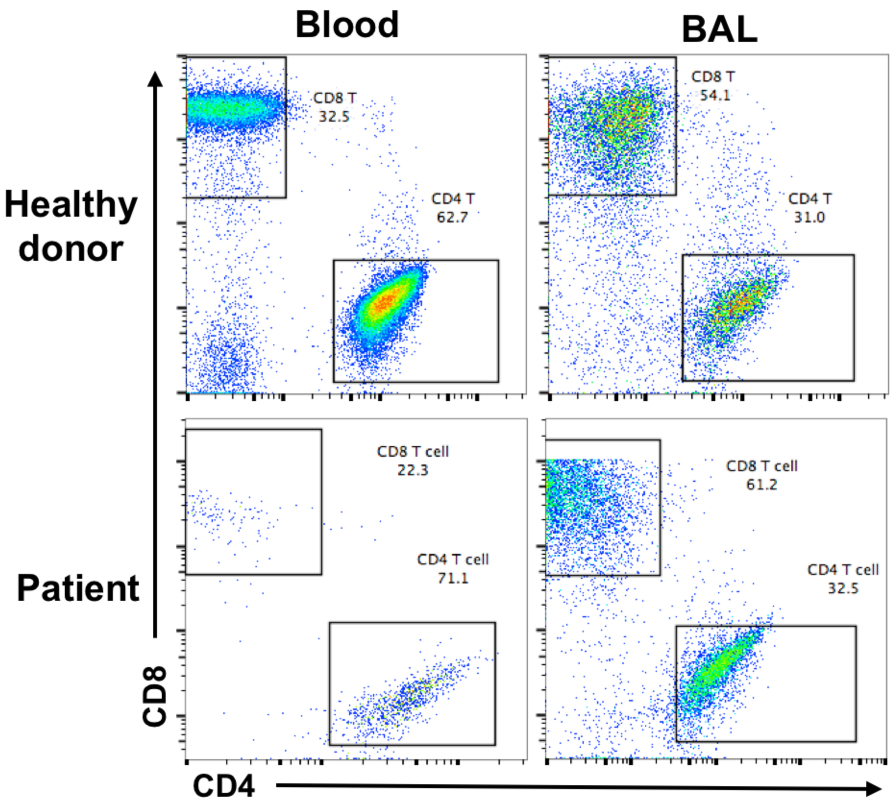

Supplement: Supplementary file 1 [file ijms-18-00616-s001.pdf]
